# Macrolide-Resistant *Mycoplasma genitalium* in Southeastern Region of the Netherlands, 2014–2017

**DOI:** 10.3201/eid2507.181556

**Published:** 2019-07

**Authors:** Liesbeth Martens, Sharon Kuster, Wilco de Vos, Maikel Kersten, Hanneke Berkhout, Ferry Hagen

**Affiliations:** Radboud University Medical Center, Nijmegen, the Netherlands (L. Martens);; Canisius-Wilhelmina Hospital, Nijmegen (L. Martens, S. Kuster, W. de Vos, M. Kersten, H. Berkhout, F. Hagen);; Westerdijk Fungal Biodiversity Institute, Utrecht, the Netherlands (F. Hagen)

**Keywords:** *Mycoplasma genitalium*, macrolide resistance, molecular diagnostics, sexually transmitted disease, bacteria, the Netherlands, antimicrobial resistance

## Abstract

*Mycoplasma genitalium* infections of the urogenital tract are usually treated with azithromycin; however, for the past several years, rates of azithromycin treatment failure have increased. To document the occurrence and frequency of macrolide resistance–mediating mutations (MRMMs) in *M. genitalium* infections, we collected 894 *M. genitalium*–positive samples during April 2014–December 2017 and retrospectively tested them for MRMMs. We designated 67 samples collected within 6 weeks after a positive result as test-of-cure samples; of these, 60 were MRMM positive. Among the remaining 827 samples, the rate of MRMM positivity rose from 22.7% in 2014 and 22.3% in 2015 to 44.4% in 2016 but decreased to 39.7% in 2017. Because of these high rates of MRMMs in *M. genitalium* infections, we recommend that clinicians perform tests of cure after treatment and that researchers further explore the clinical consequences of this infection.

Since *Mycoplasma genitalium* was first isolated from men with nongonococcal urethritis in 1981, this bacterium has been recognized as a possible pathogen of the genitourinary tract (*1*). During the first decade after its discovery, its fastidious nature and slow growth rate complicated research into its clinical significance. Its association with nongonococcal urethritis in men was not established until the mid-1990s (*2*,*3*), when molecular testing in research settings had become available. Now *M. genitalium* is widely recognized as a frequent cause of male urethritis (*4*,*5*), a likely cause of cervicitis and pelvic inflammatory disease in women (*6*), and a possible cause of several other genitourinary syndromes (*4*–*7*).

The commonly used treatment for *M.* *genitalium* infections is azithromycin, either in a single dose of 1,000 mg or as a 5-day regimen (500 mg on the first day, followed by 250 mg on the subsequent 4 days). During the past decade, azithromycin treatment failure has been reported with increasing frequency (*8*). Clinical cure rates reported before 2008 were generally >80% (*9*–*11*) but more recently have dropped to as low as 54% (*12*,*13*). A single dose of azithromycin is the preferred treatment for nongonococcal urethritis in many countries, including Australia (*14*), the Netherlands (*15*), the United States (*16*), and the United Kingdom (*17*). Moreover, *Chlamydia trachomatis* infections are also commonly treated with a single dose of azithromycin, often without excluding co-infection with *M. genitalium* (*14*–*17*). However, it has been suggested that the single-dose regimen of azithromycin is actually facilitating the development of macrolide resistance (*18*,*19*).

In the Netherlands, routine testing for *M. genitalium* is not included in national sexually transmitted disease (STD) screening protocols (*15*,*20*,*21*). In a recent revision of the national protocol, screening for *M. genitalium* infections in men with nongonococcal urethritis is mentioned but not explicitly advised (*22*). The protocol states that the treatment of choice for symptomatic men and their partners is azithromycin for 5 days or moxifloxacin for 7–10 days. Follow-up is not mentioned (*22*). Most local hospital-based guidelines advise azithromycin in a 5-day regimen only; some advise a test of cure. Detection of macrolide resistance–mediating mutations (MRMMs) has, so far, not been included as part of the diagnostic work-up.

To document the occurrence and frequency of MRMMs, we conducted a retrospective study in the Netherlands during April 2014–December 2017. We tested samples that were positive for *M. genitalium* during the study period for MRMMs by using a molecular diagnostic approach.

## Material and Methods

### Sampling

The Laboratory of Medical Microbiology of the Canisius-Wilhelmina Hospital in Nijmegen, the Netherlands, is the primary diagnostic laboratory for the hospital itself, other care institutions, and general practitioners in the area. All samples referred to this laboratory for STD diagnostics starting in April 2014 were tested for *M. genitalium*, and samples that were positive were stored at −80°C. The study was approved by the Canisius-Wilhelmina Hospital Institution Review Board. Because patient identities were anonymous, written informed consent of participants was not required.

### DNA Extraction

We collected *M. genitalium*–positive samples (determined by routine diagnostic in-house quantitative PCR [qPCR]) from the −80°C storage. After thawing the samples, we homogenized them by short vortexing and subsequently mixed a 200-μL sample with 20 μL internal control (phocine herpes virus [PhHV]) into a 96–deep well plate and extracted nucleic acids by using the MagNA Pure 96 automatic platform (Roche, http://www.roche.com). Nucleic acids eluates were directly subjected to molecular testing and stored at −20°C until further use.

### Detection of STDs 

We again subjected all samples to the same routine diagnostic in-house qPCR that simultaneously detects *Trichomonas vaginalis*, *M. genitalium*, and the internal extraction and amplification control PhHV. The qPCR mixture consisted of 2 μL primers and probes MgeFwd (5′-GAGAAATACCTTGATGGTCAGCAA-3′, 5 pmol/μL), MgeRvd (5′-GTTAATATCATATAAAGCTCTACCGTTGTTATC-3′, 5 pmol/μL), MgeProbe (5′-FAM-ACTTTGCAATCAGAAGGT-MGB-3′, 2 pmol/μL), TvaFwd (5′-CCTCAGTTCGCAAAGGC-3′, 5 pmol/μL), TvaRvd (5′-TTCAGCGACCATTCCCA-3′, 5 pmol/μL), TvaProbe (5′-HEX-CATTGACGCACTCATGACGAACGA-BHQ1-3′, 2 pmol/μL), PhHVFwd (5′-GGGCGAATCACAGATTGAATC-3′, 5 pmol/μL), PhHVRvd (5′-GCGGTTCCAAACGTACCAA-3′, 5 pmol/μL), and PhHVProbe (5′-Cy5-TTTTTATGTGTCCGCCACCATCTGGATC-BHQ2-3′, 2 pmol/μL) (*23*); 10 μL 2× Fast Advanced Master Mix (Applied Biosystems, https://www.thermofisher.com); and 8 μL DNA sample. The qPCR reaction was conducted in a LightCycler 480-II (Roche Diagnostics) with the following protocol: UNG (uracil-*N*-glycosylase) treatment for 2 min at 50°C, 10 min polymerase activation at 96°C, 45 cycles of denaturation for 5 s at 96°C and 12 s at 60°C (fluorescence measurement), and a final cooling step for 30 s at 40°C. We included a negative extraction control, a negative template control, and a positive template control.

### Detection of *M. genitalium* and MRMMs 

The TVMGres qPCR assay developed by NYtor (https://www.nytor) is a multiplex qPCR assay that detects *T. vaginalis* and *M. genitalium* and includes the internal control PhHV. The assay simultaneously detects the single-nucleotide polymorphisms A2058C, A2058G, A2058T, and A2059G in the 23S ribosomal RNA–encoding region of *M. genitalium,* which together account for >95% of the cases of azithromycin resistance (*24*). We added 5 μL of the nucleic acids extract to 15-μL reaction mix that contained qPCR master mix and the primer/probe mix from the TVMGres qPCR assay. We performed the qPCR reactions on a LightCycler 480-II instrument (Roche Diagnostics) using a reaction protocol consisting of a polymerase activation step of 3 min at 95°C, followed by 45 cycles of 15 s at 95°C and 1 min at 60°C. Detection was done by measuring the fluorescence signals of FAM (for *T. vaginalis*), CAL Fluor Orange 560 (for *M. genitalium* mutations), CAL Fluor Red 610 (for *M. genitalium* detection), and TYE665 (for internal control) (https://eu.idtdna.com for all dyes). We considered a sample to be valid if an amplification curve for any of the pathogen targets or the internal control was present.

We defined wild-type *M. genitalium* as that in a sample that was positive for *M. genitalium* without the presence of a positive MRMM signal. We defined resistant *M. genitalium* as that in a sample positive for both *M. genitalium* and MRMMs.

### Statistical Analyses

We collected data in Microsoft Excel (Microsoft, https://www.microsoft.com) and used Microsoft Office Excel and SPSS (IBM, https://www.ibm.com) for analysis. We calculated p values in SPSS by using the χ^2^ test and 2-sample *t*-test.

## Results

We tested 28,408 samples from 20,537 patients for the presence of STD organisms. Most (n = 25,132; 88.5%) samples were provided by general practitioners, 3,087 (10.9%) by hospitals, and 189 (0.7%) from other and unknown locations. *M. genitalium* was detected in 1,146 (4.0%) samples from 879 patients (4.3%). For 7 patients, multiple samples that were collected on the same day were positive; for each of these patients, we included only 1 of these samples, which left 1,139 samples, 936 of which were available for further testing. The remaining 203 samples were either not stored or were of inadequate volume for further analysis. We found no statistically significant differences between available and unavailable samples (Table).

**Table:** Characteristics of samples available and unavailable for further testing in study of macrolide-resistant *Mycoplasma genitalium* in southeastern region of the Netherlands, 2014–2017*

Characteristic	Available samples, % (n = 936)	Unavailable samples, % (n = 203)	p value
Patient sex			
F	67.3	69.5	0.546
M	32.7	30.5	
Proportion sent in by general practitioner	92.2	90.2	0.343
Proportion test of cure	7.3	8.9	0.357

*Average ages: patients for whom samples were available, 29.8 ± 9.3 y; patients for whom samples were not available, 30.9 ± 9.5 y; p = 0.118.

**Figure 1:**
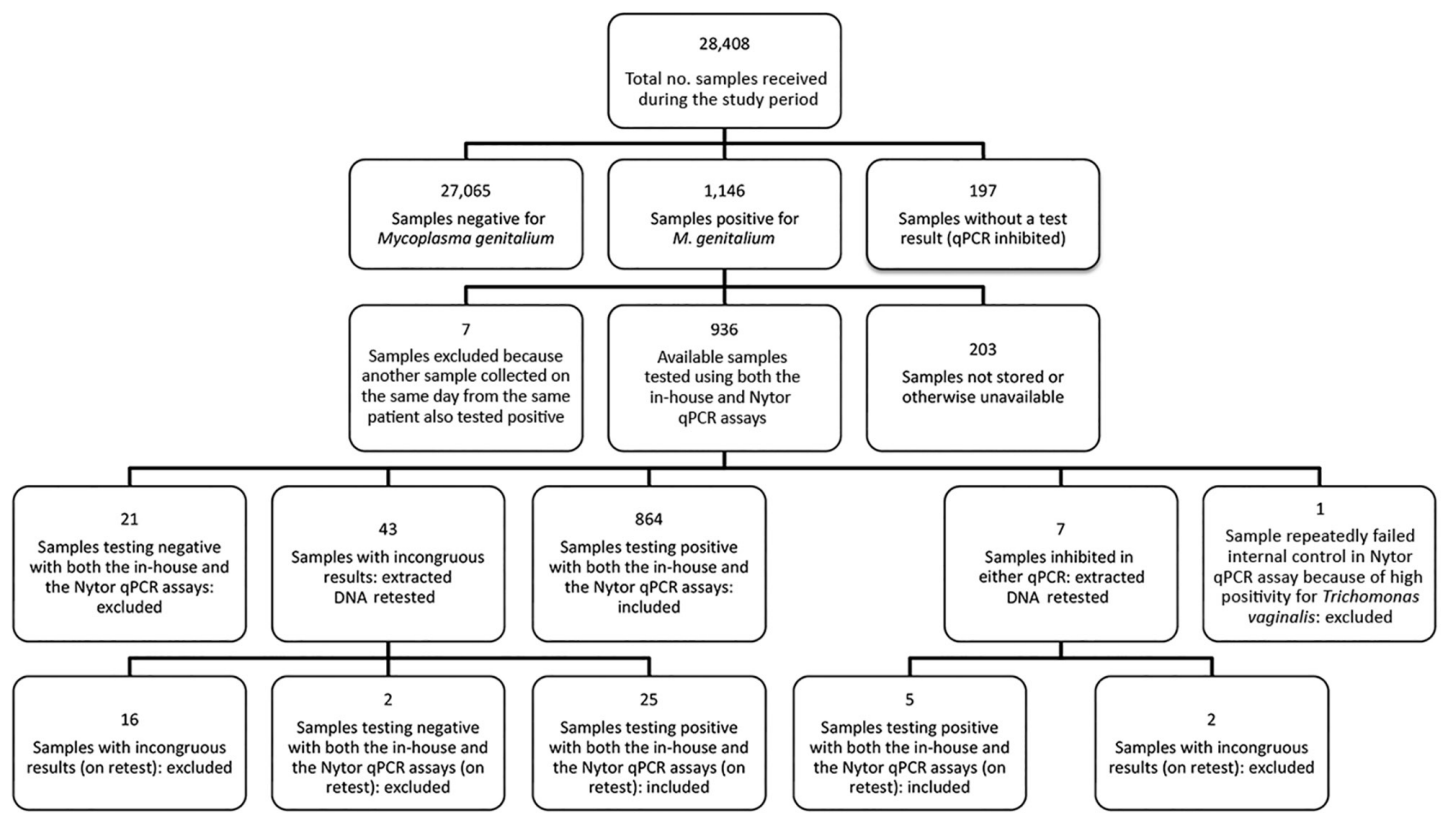
Characteristics of all samples received during study of macrolide-resistant *Mycoplasma genitalium* in southeastern region of the Netherlands, 2014–2017. qPCR, quantitative PCR.

A total of 23 samples were negative for *M. genitalium* by the routine diagnostic in-house qPCR and the TVMGres qPCR assay. We excluded these samples from our study, together with 18 samples repeatedly showing incongruous results (i.e., in-house qPCR result not matching the TVMGres qPCR result). We also excluded another sample that repeatedly had an inhibited internal control in the TVMGres qPCR assay because of a concurrent *T. vaginalis* infection. We provide an overview of all included and excluded samples (Figure 1).

### Characteristics of the Study Population

Of the 28,408 samples that were tested for *M. genitalium* during the study period, 19,393 (68.3%) originated from female patients and 9,015 (31.7%) from male patients (2.2:1 ratio). The percentages of *M. genitalium* positivity were similar for male (4.1%) and female (4.0%) patients.

Average age at the time of testing was 32.0 years; female patients (average 31.3 years of age) were younger than male patients (average 33.6 years of age). The average age of *M. genitalium*–positive male patients (33.1 years) was slightly lower than that of *M. genitalium*–negative male patients (33.6 years; p = 0.36). *M. genitalium*–positive female patients were 2.8 years younger than *M. genitalium*–negative female patients (28.5 vs. 31.3 years; p<0.001).

### Epidemiology

**Figure 2:**
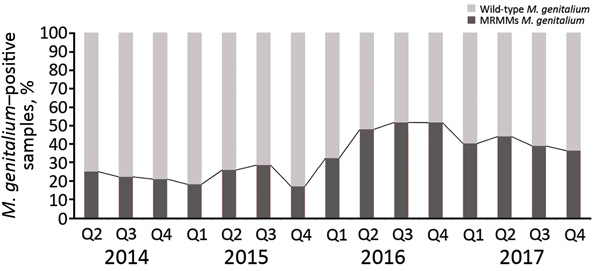
Proportion of *Mycoplasma genitalium*–positive samples containing MRMMs in study of macrolide-resistant *M. genitalium* in southeastern region of the Netherlands, 2014–2017. Percentages of *M. genitalium*–positive samples that are either wild-type or positive for MRMMs are based on quantitative PCR. MRMM, macrolide resistance–mediating mutation; Q, quarter.

A total of 894 samples had positive results for *M. genitalium* when tested by the routine diagnostic in-house qPCR and by the TVMGres qPCR assay. Of these, 67 (7.5%) samples were collected within 6 weeks of a previous positive *M. genitalium* result. These retests were considered positive results for tests of cure, which were analyzed separately. Of the remaining 827 samples, 281 (34.0%) were *M. genitalium* MRMM–positive by qPCR. The frequency of MRMMs almost doubled from 22.7% (27/119) in 2014 and 22.3% (47/211) in 2015 to 44.4% (92/207) in 2016, then decreased to 39.7% (115/290) in 2017 (Figure 2).

### Tests of Cure

**Figure 3:**
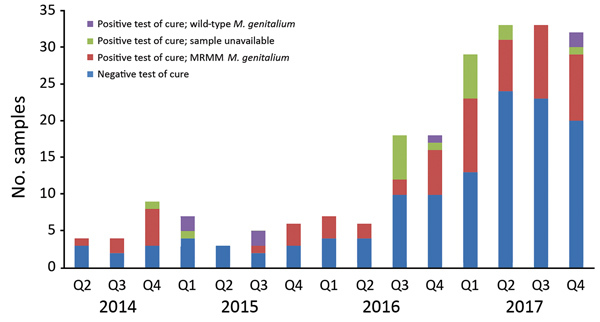
Characteristics of test-of-cure samples in study of macrolide-resistant *Mycoplasma genitalium* in southeastern region of the Netherlands, 2014–2017. Positive results indicate presence of *M. genitalium*; negative results indicate no *M. genitalium*. Q, quarter.

**Figure 4:**
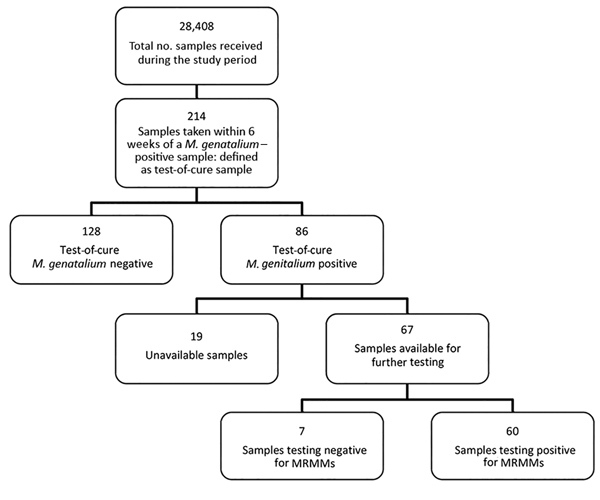
Identification and test results for test-of-cure samples in study of macrolide-resistant *Mycoplasma genitalium* in southeastern region of the Netherlands, 2014–2017. Positive results indicate presence of *M. genitalium*; negative results indicate no *M. genitalium*. MRMM, macrolide resistance–mediating mutation.

Samples taken from a patient within 6 weeks of an *M. genitalium*–positive sample were defined as test-of-cure samples. During the study period, we collected 214 samples for test of cure, most (163; 76.2%) from the second half of 2016 on. Of the 214 samples, 86 (40.2%) were *M. genitalium* positive; of these, 67 (77.9%) were available for further testing. Only 7 (10.4%) samples were found to contain wild-type *M. genitalium* (Figures 3, 4).

For 57 positive test-of-cure samples, both the initial sample and the test(s)-of-cure samples were available. In most instances, both samples were positive for MRMMs (45/57; 78.9%).

Seven patients who were originally infected with a wild-type strain had resistant *M. genitalium* in the test-of-cure sample. For 1 patient, the initial sample was positive for MRMMs but the test-of-cure sample, collected 41 days later, contained wild-type *M. genitalium*. For the remaining 4 patients, both the initial sample and the test-of-cure sample contained wild-type *M. genitalium*.

## Discussion

This retrospective epidemiologic study shows that the frequency of MRMMs in *M. genitalium* in the southeastern region of the Netherlands almost doubled from 2014 and 2015 to 2016. After 2016, the frequency of MRMMs gradually decreased through the last quarter of 2017.

High MRMM frequency in *M. genitalium* has been described before (*25*–*36*) and is thought to be a consequence of using the single-dose azithromycin treatment regimen for *C. trachomatis* infections without excluding the presence of *M. genitalium* nongonococcal urethritis in men, *M. genitalium* infections, or both (*18*,*19*). To our knowledge, the decreasing number of MRMMs observed in *M. genitalium* from our study population in 2017 compared with 2016 has not been described before; however, compared with 2014 and 2015, the MRMM prevalence trend is increasing. During the study period, local protocols for the treatment of the aforementioned conditions were unchanged; however, starting on October 3, 2016, the advice to perform a test of cure 3 weeks after treatment was added in print to every report of a positive *M. genitalium* test result. In the preceding months, the advice was often given on an individual basis. Thus, in the second half of 2016, the number of tests of cure increased sharply (Figure 3). This increased testing may help explain the decrease in MRMM frequency because the 40.2% with a test-of-cure result that was positive for *M. genitalium* would logically have been subsequently treated with a different treatment regimen. However, even in 2017, the year in which most tests of cure were conducted, only about one third of the positive *M. genitalium* samples were followed up by a test of cure. Regardless whether the decreased MRMM frequency resulted from the increased number of tests of cure, the high rate of MRMMs in itself calls for a test of cure after treatment with azithromycin.

Most positive test-of-cure samples (60/67; 89.6%) contained MRMMs. This finding was expected because given that azithromycin is the preferred treatment for *M. genitalium* according to local protocol, macrolide-susceptible strains would have resulted in a negative test-of-cure result. Of note, for 7 patients who were originally infected with a wild type strain, test-of-cure results indicated a resistant *M. genitalium* strain. One patient with a resistant strain turned out to have a wild-type strain 6 weeks later; however, because it is unknown what treatment these patients received and whether they were at risk of acquiring a second *M. genitalium* infection, we cannot comment on the reason. The 4 patients with a persistent wild-type *M. genitalium* infection may have been reinfected, may have had a resistant *M. genitalium* infection that cannot be detected by the qPCR, may have been prescribed a nonmacrolide drug (e.g., doxycycline), or may have neglected to take the prescribed treatment.

A limitation of our study is the number of samples unavailable for further testing, which was 203 (17.8%) of 1,139. The reasons were generally either the sample not being stored or the volume of the stored sample being too low. In the third quarter of 2016, a disproportionately large number of samples (52/83; 62.7%) was lost because of a misunderstanding in storage protocol (i.e., which samples should and should not be stored). Unfortunately, this period coincided with a peak of MRMM frequency (17/31; 54.8%). However, in the fourth quarter of 2016, only 9/81 (11.1%) of samples were lost and MRMM frequency was the same (38/69; 55.1%). Statistically, we found no difference in characteristics of the populations for whom samples were available or unavailable (Table).

The question whether asymptomatic persons should be screened for *M. genitalium* can only be answered with more research into the clinical aspects and (long-term) complications of (co-)infection with *M. genitalium*. However, if a single dose of azithromycin indeed predisposes toward the development of MRMMs, *M. genitalium* infection should be excluded before *C. trachomatis* infections are treated with this regimen. In addition, treatment of nongonococcal urethritis should be reevaluated.

In conclusion, high rates (up to 44.4%) of MRMMs in *M. genitalium* were found among patients from the southeastern region of the Netherlands who were screened for STDs during 2014–2017. After a sharp initial increase, MRMM prevalence among *M. genitalium*–positive samples declined from the first quarter of 2017 on. This finding may or may not be a consequence of the increased number of tests of cure performed during the months immediately preceding the decline. However, regardless of the reason for the decline, we believe that the rates of MRMMs in *M. genitalium* call for a recommendation to perform a test of cure after treatment of *M. genitalium* infections.

## References

[1] Tully JG, Taylor-Robinson D, Cole RM, Rose DL. A newly discovered *mycoplasma* in the human urogenital tract. Lancet. 1981;1:1288–91. 10.1016/S0140-6736(81)92461-2 6112607

[2] Horner PJ, Gilroy CB, Thomas BJ, Naidoo RO, Taylor-Robinson D. Association of *Mycoplasma genitalium* with acute non-gonococcal urethritis. Lancet. 1993;342:582–5. 10.1016/0140-6736(93)91411-E 8102721

[3] Jensen JS, Orsum R, Dohn B, Uldum S, Worm AM, Lind K. *Mycoplasma genitalium*: a cause of male urethritis? Genitourin Med. 1993;69:265–9. 10.1136/sti.69.4.265 7721285 PMC1195084

[4] Horner PJ, Martin DH. *Mycoplasma genitalium* infection in men. J Infect Dis. 2017;216(suppl_2):S396–405. 10.1093/infdis/jix145 PMC5853510 28838074

[5] Bradshaw CS, Jensen JS, Tabrizi SN, Read TR, Garland SM, Hopkins CA, et al. Azithromycin failure in *Mycoplasma genitalium* urethritis. Emerg Infect Dis. 2006;12:1149–52. 10.3201/eid1207.051558 16836839 PMC3291056

[6] Wiesenfeld HC, Manhart LE. *Mycoplasma genitalium* in women: current knowledge and research priorities for this recently emerged pathogen. J Infect Dis. 2017;216(suppl_2):S389–95. 10.1093/infdis/jix198 PMC5853983 28838078

[7] Taylor-Robinson D, Jensen JS. *Mycoplasma genitalium*: from Chrysalis to multicolored butterfly. Clin Microbiol Rev. 2011;24:498–514. 10.1128/CMR.00006-11 21734246 PMC3131060

[8] Manhart LE, Jensen JS, Bradshaw CS, Golden MR, Martin DH. Efficacy of antimicrobial therapy for *Mycoplasma genitalium* infections. Clin Infect Dis. 2015;61(Suppl 8):S802–17. 10.1093/cid/civ785 26602619

[9] Gambini D, Decleva I, Lupica L, Ghislanzoni M, Cusini M, Alessi E. *Mycoplasma genitalium* in males with nongonococcal urethritis: prevalence and clinical efficacy of eradication. Sex Transm Dis. 2000;27:226–9. 10.1097/00007435-200004000-00008 10782745

[10] Wikström A, Jensen JS. *Mycoplasma genitalium*: a common cause of persistent urethritis among men treated with doxycycline. Sex Transm Infect. 2006;82:276–9. 10.1136/sti.2005.018598 16877573 PMC2564707

[11] Stamm WE, Batteiger BE, McCormack WM, Totten PA, Sternlicht A, Kivel NM; Rifalazil Study Group. A randomized, double-blind study comparing single-dose rifalazil with single-dose azithromycin for the empirical treatment of nongonococcal urethritis in men. Sex Transm Dis. 2007;34:545–52. 10.1097/01.olq.0000253348.44308.8c 17297383

[12] Manhart LE, Gillespie CW, Lowens MS, Khosropour CM, Colombara DV, Golden MR, et al. Standard treatment regimens for nongonococcal urethritis have similar but declining cure rates: a randomized controlled trial. Clin Infect Dis. 2013;56:934–42. 10.1093/cid/cis1022 23223595 PMC3588116

[13] Anagrius C, Loré B, Jensen JS. Treatment of *Mycoplasma genitalium*. Observations from a Swedish STD clinic. PLoS One. 2013;8:e61481. 10.1371/journal.pone.0061481 23593483 PMC3620223

[14] Australasian Sexual Health Alliance. Guideline on urethritis [cited 2018 Oct 5]. http://www.sti.guidelines.org.au/syndromes/urethritis-male#management

[15] De Vries HJC, Van Doornum GJJ. Toelichting bij de nieuwe samenvattingskaart 2014/2015. Diagnostiek en behandeling van seksueel overdraagbare aandoeningen. Tijdschrift voor Infectieziekten. 2014;9:9–11.

[16] Centers for Disease Control and Prevention. Sexually transmitted diseases treatment guidelines [cited 2018 Oct 5]. https://www.cdc.gov/std/tg2015/default.htm

[17] British Association for Sexual Health and HIV. Guideline on NGU [cited 2018 Oct 1]. https://www.bashh.org/guidelines

[18] Jensen JS, Bradshaw CS, Tabrizi SN, Fairley CK, Hamasuna R. Azithromycin treatment failure in *Mycoplasma genitalium*-positive patients with nongonococcal urethritis is associated with induced macrolide resistance. Clin Infect Dis. 2008;47:1546–53. 10.1086/593188 18990060

[19] Horner P, Ingle SM, Garrett F, Blee K, Kong F, Muir P, et al. Which azithromycin regimen should be used for treating *Mycoplasma genitalium*? A meta-analysis. Sex Transm Infect. 2018;94:14–20. 10.1136/sextrans-2016-053060 28717050

[20] Netherlands Federation of General Physicians. Guideline on sexually transmitted infections [in Dutch] [cited 2018 Oct 5]. https://www.nhg.org/standaarden/volledig/nhg-standaard-het-soa-consult

[21] National Institute for Public Health and the Environment. Guideline on sexually transmitted infections in government-funded STI-clinics [in Dutch] [cited 2018 Oct 5]. https://lci.rivm.nl/draaiboeken/consult-seksuele-gezondheid

[22] Netherlands Association of Dermatology and Venereology. Multidisciplinary guideline on diagnostics and treatment of sexually transmitted infections [in Dutch] [cited 2018 Oct 5]. http://www.nvdv.nl/wp-content/uploads/2014/08/Definitieve-multidisciplinaire-richtlijn-soa-herziening-2018.pdf

[23] Jensen JS, Björnelius E, Dohn B, Lidbrink P. Use of TaqMan 5′ nuclease real-time PCR for quantitative detection of *Mycoplasma genitalium* DNA in males with and without urethritis who were attendees at a sexually transmitted disease clinic. J Clin Microbiol. 2004;42:683–92. 10.1128/JCM.42.2.683-692.2004 14766837 PMC344445

[24] Twin J, Jensen JS, Bradshaw CS, Garland SM, Fairley CK, Min LY, et al. Transmission and selection of macrolide resistant *Mycoplasma genitalium* infections detected by rapid high resolution melt analysis. PLoS One. 2012;7:e35593. 10.1371/journal.pone.0035593 22532861 PMC3331984

[25] Anderson T, Coughlan E, Werno A. *Mycoplasma genitalium* macrolide and fluoroquinolone resistance detection and clinical implications in a selected cohort in New Zealand. J Clin Microbiol. 2017;55:3242–8. 10.1128/JCM.01087-17 28878004 PMC5654908

[26] Barberá MJ, Fernández-Huerta M, Jensen JS, Caballero E, Andreu A. *Mycoplasma genitalium* macrolide and fluoroquinolone resistance: prevalence and risk factors among a 2013–2014 cohort of patients in Barcelona, Spain. Sex Transm Dis. 2017;44:457–62. 10.1097/OLQ.0000000000000631 28703723

[27] Braam JF, Slotboom B, Van Marm S, Severs TT, Van Maarseveen NM, Van Zwet T, et al. High prevalence of the A2058T macrolide resistance-associated mutation in *Mycoplasma genitalium* strains from the Netherlands. J Antimicrob Chemother. 2017;72:1529–30. 10.1093/jac/dkw584 28158595

[28] Couldwell DL, Jalocon D, Power M, Jeoffreys NJ, Chen SCA, Lewis DA. *Mycoplasma genitalium*: high prevalence of resistance to macrolides and frequent anorectal infection in men who have sex with men in western Sydney. Sex Transm Infect. 2018;94:406–10. 10.1136/sextrans-2017-053480 29567802

[29] Gossé M, Lysvand H, Pukstad B, Nordbø SA. A novel SimpleProbe PCR assay for detection of mutations in the 23S rRNA gene associated with macrolide resistance in *Mycoplasma genitalium* in clinical samples. J Clin Microbiol. 2016;54:2563–7. 10.1128/JCM.01233-16 27487958 PMC5035408

[30] Gratrix J, Plitt S, Turnbull L, Smyczek P, Brandley J, Scarrott R, et al. Prevalence and antibiotic resistance of *Mycoplasma genitalium* among STI clinic attendees in Western Canada: a cross-sectional analysis. BMJ Open. 2017;7:e016300. 10.1136/bmjopen-2017-016300 28698342 PMC5541599

[31] Nijhuis RH, Severs TT, Van der Vegt DS, Van Zwet AA, Kusters JG. High levels of macrolide resistance-associated mutations in *Mycoplasma genitalium* warrant antibiotic susceptibility-guided treatment. J Antimicrob Chemother. 2015;70:2515–8. 10.1093/jac/dkv136 25995292

[32] Read TRH, Fairley CK, Tabrizi SN, Bissessor M, Vodstrcil L, Chow EPF, et al. Azithromycin 1.5 g over 5 days compared to 1 g single dose in urethral *Mycoplasma genitalium*: impact on treatment outcome and resistance. Clin Infect Dis. 2017;64:250–6. 10.1093/cid/ciw719 28011607

[33] Salado-Rasmussen K, Jensen JS. *Mycoplasma genitalium* testing pattern and macrolide resistance: a Danish nationwide retrospective survey. Clin Infect Dis. 2014;59:24–30. 10.1093/cid/ciu217 24729494 PMC4305131

[34] Tabrizi SN, Su J, Bradshaw CS, Fairley CK, Walker S, Tan LY, et al. Prospective evaluation of ResistancePlus MG, a new multiplex quantitative PCR assay for detection of *Mycoplasma genitalium* and macrolide resistance. J Clin Microbiol. 2017;55:1915–9. 10.1128/JCM.02312-16 28381611 PMC5442548

[35] Tagg KA, Jeoffreys NJ, Couldwell DL, Donald JA, Gilbert GL. Fluoroquinolone and macrolide resistance-associated mutations in *Mycoplasma genitalium.* J Clin Microbiol. 2013;51:2245–9. 10.1128/JCM.00495-13 23658265 PMC3697725

[36] Trembizki E, Buckley C, Bletchly C, Nimmo GR, Whiley DM. High levels of macrolide-resistant *Mycoplasma genitalium* in Queensland, Australia. J Med Microbiol. 2017;66:1451–3. 10.1099/jmm.0.000584 28893363 PMC5845567

